# Dynamic simulation of continuous mixed sugar fermentation with increasing cell retention time for lactic acid production using *Enterococcus mundtii* QU 25

**DOI:** 10.1186/s13068-020-01752-6

**Published:** 2020-06-26

**Authors:** Ying Wang, Ka-Lai Chan, Mohamed Ali Abdel-Rahman, Kenji Sonomoto, Shao-Yuan Leu

**Affiliations:** 1 Department of Biological Science, College of Life Sciences, Sichuan Normal University, Chengdu, 610101 Sichuan China; 2 Department of Civil and Environmental Engineering, Hong Kong Polytechnic University, Kowloon, Hong Kong; 3 Laboratory of Microbial Technology, Division of Systems Bioengineering, Department of Bioscience and Biotechnology, Faculty of Agriculture, Graduate School, Kyushu University, Motooka, Nishi‐ku, Fukuoka, Japan; 4 Botany and Microbiology Department, Faculty of Science (Boys), Al-Azhar University, PN:11884, Nasr City, Cairo, Egypt

**Keywords:** Lactic acid fermentation, Dilution rate, Continuous, Cell recycle, Modeling

## Abstract

**Background:**

The simultaneous and effective conversion of both pentose and hexose in fermentation is a critical and challenging task toward the lignocellulosic economy. This study aims to investigate the feasibility of an innovative co-fermentation process featuring with a cell recycling unit (CF/CR) for mixed sugar utilization. A l-lactic acid-producing strain *Enterococcus mundtii* QU 25 was applied in the continuous fermentation process, and the mixed sugars were utilized at different productivities after the flowing conditions were changed. A mathematical model was constructed with the experiments to optimize the biological process and clarify the cell metabolism through kinetics analysis. The structured model, kinetic parameters, and achievement of the fermentation strategy shall provide new insights toward whole sugar fermentation via real-time monitoring for process control and optimization.

**Results:**

Significant carbon catabolite repression in co-fermentation using a glucose/xylose mixture was overcome by replacing glucose with cellobiose, and the ratio of consumed pentose to consumed hexose increased significantly from 0.096 to 0.461 by mass. An outstanding product concentration of 65.2 g L^−1^ and productivity of 13.03 g L^−1^ h^−1^ were achieved with 50 g L^−1^ cellobiose and 30 g L^−1^ xylose at an optimized dilution rate of 0.2 h^−1^, and the cell retention time gradually increased. Among the total lactic acid production, xylose contributed to more than 34% of the mixed sugars, which was close to the related contents in agricultural residuals. The model successfully simulated the transition of sugar consumption, cell growth, and lactic acid production among the batch, continuous process, and CF/CR systems.

**Conclusion:**

Cell retention time played a critical role in balancing pentose and hexose consumption, cell decay, and lactic acid production in the CF/CR process. With increasing cell concentration, consumption of mixed sugars increased with the productivity of the final product; hence, the impact of substrate inhibition was reduced. With the validated parameters, the model showed the highest accuracy simulating the CF/CR process, and significantly longer cell retention times compared to hydraulic retention time were tested.

## Background

Lignocellulosic biomass in the form of agricultural residues and urban wastes represents a near-term solution for the production of biofuel and biochemicals to alleviate global climate change. Optically pure lactic acid (LA) production through fermentation has attracted increasing attention for the manufacture of polylactic acid (PLA), a biodegradable and biocompatible polymer, as a substitute for petrochemical-derived plastics [1]. Lactic acid fermentation using starch-based feedstock has been well established and commercialized, but this approach has been challenged due to the well-known competition of food vs. fuel [2] and high feedstock costs [3]. Applying lignocellulosic biomass for lactic acid production is environmentally preferred, but is also a challenging task due to the increasing complexity of treatment processes, i.e., pretreatment [4], saccharification [5], and fermentation [6].

Simultaneous and efficient utilization of hexoses and pentoses derived from lignocellulosic biomass is a critical bottleneck to the complete utilization of substrates [7]. Lignocellulosic hydrolysates are composed of 50–70% cellodextrins and glucose, 20–30% xylose, 0–25% lignin, some extractives (such as terpenes alkaloids, fats, and waxes), and reaction by-products, of which the types and proportion of sugars vary significantly among biomass and pretreatment methods [8, 9]. Current biorefinery processes have successfully applied hexoses for lactic acid fermentation [10], but utilization of pentoses is still challenging due to the metabolic regulation of carbon catabolite repression (CCR) [11]. While many strains have been discovered or engineered to use pentoses for fermentation, both natural and engineered microorganisms tend to utilize glucose preferentially when mixed sugars are provided in the process. When the glucose concentration is higher than a certain threshold, the less preferred sugars, such as xylose, may not be utilized by fermentation microorganisms, and hence are left in the fermentation broth even after the glucose is completely utilized.

Significant research efforts have been made to overcome the glucose-induced CCR in mixed sugar fermentation. Taniguchi et al. [12] developed a co-cultivation process with a two-stage cultivation using two lactic acid-producing bacteria specific for glucose and xylose, and produced a high amount of lactic acid (95 g L^−1^) from 100 g L^−1^ glucose and 50 g L^−1^ xylose (G100X50). However, no improvement in xylose consumption was achieved. Lu et al. [13] introduced an engineered *Escherichia coli* strain, JH15, in the production of d-lactic acid and obtained 83 g L^−1^ lactic acid from G50X50. The strain indeed consumed some xylose but the overall lactic acid productivity decreased to 0.87 g L^−1^ h^−1^ compared with 2.44 g L^−1^ h^−1^ using 100 g L^−1^ glucose as the substrate. Another strategy to overcome CCR is through careful adjustment of the feeding sugar combination in the fermentation process. Wang et al. [14] discovered that complete utilization of xylose can be achieved by replacing glucose with cellobiose without inducing the CCR. In a fed-batch fermentation system with C100X60, up to 163 g L^−1^ lactic acid was produced using a xylose consuming strain (i.e., *Enterococcus mundtii* QU 25, the strain used in this study), which is subjected to the CCR when isolated and characterized [15]. However, the productivity of 0.68 g L^−1^ h^−1^ in this process was not outstanding, and can be further improved through advanced biological processes and bioreactor systems.

Continuous fermentation associated with cell recycling has shown many superior features, such as increased productivity of bio-products over batch- or fed-batch processes [16]. The operational benefits of the continuous process are high production rate and reduced downtime for cleaning, filling and sanitation [17]. By controlling the cell concentration and/or the cell retention time (CRT) of the process, the robustness of the continuous process can be improved to handle different substrate combinations with a higher variety of digestibilities or toxicities. The process can be operated without repeating the inoculation and may prevent significant cell decay due to sudden environmental changes [18], which may be applied to overcome the CCR and/or growth-inhibiting effects induced by pretreatment by-products, carbon sources, and end-products.

**Fig. 1:**
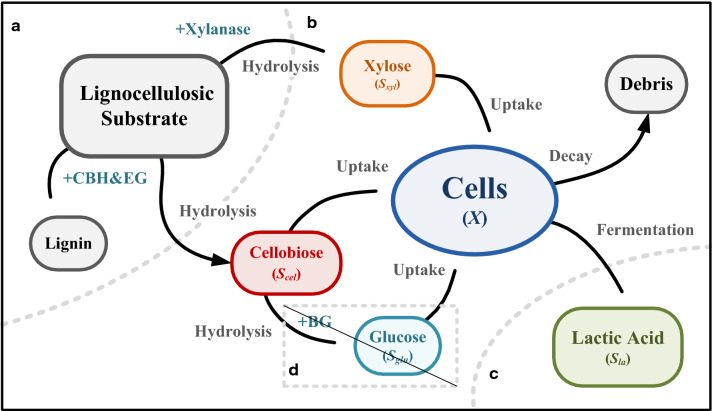
Conceptual diagram and model structure of the continuous lactic acid co-fermentation process with membrane separation and selected enzyme combination for preventing carbon catabolite repression; **a** hydrolysis system; **b** fermentation; **c** membrane separation; and **d** pathway to be cut-off to prevent carbon catabolite repression (CCR)

In this study, the potential benefits of cellobiose/xylose co-fermentation were investigated in a continuous fermentation system with a cell recycling unit and were supported by dynamic modeling. The basic concept of bioconversion process and model structure is shown in Fig. 1. To better elucidate the metabolic kinetics of the fermentation strain in the specific bioreactor system, synthetic hydrolysates were applied to simulate a staged hydrolysis with reduced glucosidase (BG), of which the hydrolysis of cellobiose to glucose was partially prohibited. This strategy is potentially feasible through the application of commercial cellulase cocktails, while the related optimization of hydrolysis on real lignocellulosic biomass is in progress. The lactic acid producer *E. mundtii* QU 25 used in this study was isolated by our co-authors Abdel-Rahman and Sonomoto et al. [19], which was introduced in greater details in the later section. A mixture of cellobiose and xylose was fed to the system with *E. mundtii* QU 25 for continuous lactic acid fermentation. A hollow fiber microfiltration module was applied to control the CRT, and separate the residual sugars and lactic acid. The dynamic model was constructed and validated with the experimental results to simulate the growth and decay kinetics of the fermenting strain in various operations, i.e., batch, continuous, and cell recycling. The sensitivity of the discussed control parameters and influent conditions were drawn to visualize the operational boundary of the overall system, and to provide insights into process control, monitoring, and optimization.

### Model development

**Fig. 2:**
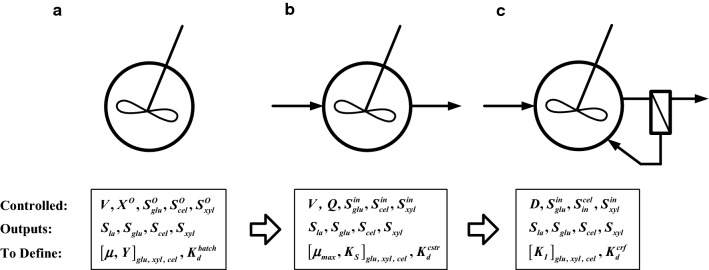
Tested and simulated systems with key model parameters; **a** batch reactor; **b** continuous flow; and **c** continuous flow with cell recycling (dilution ratio (*D*) is the inverse of cell retention time). Controlled parameters described the experimental design; growth kinetics is given after references or model validation; outputs are simulated data

The configuration of the bioreactor operations and model development and validation in various process modes are illustrated in Fig. 2. The model parameters, such as the growth kinetics, were validated by seeking for the least differentials between the simulation results and experimental data. The model was applied to simulate the biological systems with increased complexity of the process conditions, i.e., from batch reactor with three single sugars; batch reactor with mixed sugars; the continuous flow stirring tank rector (CFSTR); and to the continuous co-fermentation with cell recycling (CF/CR). The following equations for sugar utilization were derived to establish the mass balance in the dynamic situations of the reagents and products in the fermentation systems.

The stoichiometry describing sugar consumption, and productions of cell and lactic acid was derived to clarify the molar yield coefficients of the studied strain. The empirical functions were constructed upon the Embden–Meyerhof–Parnas (EMP) pathway and the pentose phosphate (PP)/glycolic pathway from hexose and pentose, respectively; or the homo-lactic acid metabolism, as detailed by Wang et al. [7]. Based on this metabolism it was assumed that no respiration (formation of CO_2_) occurred from the simulated carbon sources. Meanwhile, the fermentation by-products (i.e., acetic acid and ethanol) through the hetero-lactic acid metabolism (i.e., phosphoketolase, or the PK pathway [7]) were negligible as confirmed in our previous study [14]. The stoichiometry of the interested carbon sources was expressed as follows:

1 $${\text{Glucose }}\left( {{\text{C}}_{6} {\text{H}}_{{12}} {\text{O}}_{6} = {\text{ }}180{\text{ g}}\,{\text{mole}}^{{ - 1}} } \right):\,{\text{C}}_{6} {\text{H}}_{{12}} {\text{O}}_{6} + Y_{{{\text{glu}}}} {\text{NH}}_{4}^{ + } \to Y_{{{\text{glu}}}} {\text{C}}_{5} {\text{H}}_{7} {\text{NO}}_{2} + \left( {2 - \frac{5}{3} \cdot Y_{{{\text{glu}}}} } \right){\text{C}}_{3} {\text{H}}_{6} {\text{O}}_{3} + 3 \cdot Y_{{{\text{glu}}}} {\text{H}}_{2} {\text{O}} + Y_{{{\text{glu}}}} {\text{H}}^{ + }$$

2 $${\text{Cellobiose }}\left( {{\text{C}}_{{12}} {\text{H}}_{{22}} {\text{O}}_{{11}} = 342{\text{ g mole}}^{{ - 1}} } \right): {\text{C}}_{ 1 2} {\text{H}}_{ 2 2} {\text{O}}_{ 1 1} + Y_{{\text{cel}}} {\text{NH}}_{ 4}^{ + } \to Y_{{\text{cel}}} {\text{C}}_{ 5} {\text{H}}_{ 7} {\text{NO}}_{ 2} + \left( {4 - \frac{5}{3} \cdot Y_{{\text{cel}}} } \right){\text{C}}_{ 3} {\text{H}}_{ 6} {\text{O}}_{ 3} + \left( {3 \cdot Y_{{\text{cel}}} - 1} \right) \cdot {\text{H}}_{ 2} {\text{O}} + Y_{{\text{cel}}} {\text{H}}^{ + } .$$

3 $${\text{Xylose}}\left( {{{\text{C}}_5}{{\text{H}}_{10}}{{\text{O}}_5} = 150\;{\text{g}}\;{\text{mol}}{{\text{e}}^{ - 1}}} \right):{{\text{C}}_5}{{\text{H}}_{10}}{{\text{O}}_5} + {Y_{xyl}}{\text{NH}}_4^ + \to {Y_{xyl}}{{\text{C}}_5}{{\text{H}}_7}{\text{N}}{{\text{O}}_2} + \frac{5}{3} \cdot \left( {1 - {Y_{xyl}}} \right){{\text{C}}_3}{{\text{H}}_6}{{\text{O}}_3} + 3 \cdot {Y_{xyl}}{{\text{H}}_2}{\text{O + }}{Y_{xyl}}{\text{H}},$$

where *Y*_glu_, *Y*_cel_, and *Y*_xyl_ are molar yields of cell production over the respective consumed substrates. It was assumed that all the substrates can be used for cell growth and lactic acid production. The molecular weight of the cell (C_5_H_7_NO_2_) is 113 g mole^−1^, and the molecular weight of lactic acid (C_3_H_6_O_3_) is 90 g mole^−1^. The mass yields (in wt%) of cell biomass (*X*) and lactic acid (*S*_la_) were calculated based on the molar ratios as the following expressions using cellobiose as an example. Glucose and xylose were calculated in similar fashions.

4 $$Y_{{\text{cel}}}^{X} = Y_{{\text{cel}}} \cdot \frac{113}{342}$$

5 $$Y_{{\text{cel}}}^{\text{la}} = \left( {3 \cdot Y_{{\text{cel}}} - 1} \right) \cdot \frac{90}{342}$$

where $Y_{{\text{cel}}}^{X}$ and $Y_{{\text{cel}}}^{\text{la}}$ are the mass yields of cell and lactic acid per unit mass cellobiose consumed (wt%), respectively.

Cell growth and lactic acid production were described by the utilization of glucose, cellobiose and xylose as substrates during single and mixed sugar fermentation. Substrates and production inhibition were observed in batch fermentation experiments with *E. mundtii* QU 25 [20, 21]. Cell growth was defined by Monod kinetics with substrate and production inhibition terms as shown in Eq. (6). The consumptions of glucose, cellobiose and xylose were described by a linear relationship with cell growth. Lactic acid formation was also associated with cell growth.

6 $$\mu^{\text{sug}} = \frac{{\mu_{\text{max} }^{\text{sug}} \cdot S_{\text{sug}} }}{{K_{S}^{\text{sug}} + S_{\text{sug}} + \frac{{S_{\text{sug}}^{2} }}{{K_{\text{is}}^{\text{sug}} }}}} \cdot \frac{{K_{\text{ip}}^{\text{la}} }}{{K_{\text{ip}}^{\text{la}} + S_{\text{la}} }}$$

where *μ*_max_ is the maximum specific growth rate of the cell (h^−1^); *K*_*S*_ is the half saturation concentration (g L^−1^); and *K*_is_ and *K*_ip_ are the inhibition coefficients of substrate and product (g L^−1^), respectively.

Sugar consumption and lactic acid production are associated with cell growth [22]. The following ordinary differential equations (ODEs) were derived to quantify the dynamic changes of the reactants and products:

Sugar (*S*_sug_) consumption:

7 $$\frac{{\text{d}S_{\text{sug}} }}{\text{d}t} = D \cdot \left( {S_{\text{sug}}^{\text{in}} - S_{\text{sug}} } \right) - \mu^{\text{sug}} \cdot X \cdot \frac{1}{{Y_{\text{sug}}^{X} }}.$$

Cell growth:

8 $$\frac{\text{d}X}{\text{d}t} = D \cdot \left( {X^{\text{in}} - X} \right) + \left[ {\mu^{\text{glu}} + \mu^{{\text{cel}}} + \mu^{\text{xyl}} - K_{D} } \right] \cdot X.$$

Lactic acid production:

9 $$\frac{{\text{d}S_{\text{la}} }}{\text{d}t} = D \cdot \left( {S_{\text{la}}^{\text{in}} - S_{\text{la}} } \right) + \left[ {\frac{{Y_{\text{glu}}^{\text{la}} }}{{Y_{\text{glu}}^{X} }} \cdot \mu^{\text{glu}} + \frac{{Y_{{\text{cel}}}^{\text{la}} }}{{Y_{{\text{cel}}}^{X} }} \cdot \mu^{{\text{cel}}} + \frac{{Y_{\text{xyl}}^{\text{la}} }}{{Y_{\text{xyl}}^{X} }} \cdot \mu^{\text{xyl}} } \right] \cdot X,$$

where *S*_sug_ is a general form of glucose (*S*_glu_), cellobiose (*S*_cel_), and xylose (*S*_xyl_), each with independent metabolic kinetics; *D* the dilution rate (h^−1^); and *K*_*D*_ the decay coefficient (h^−1^). A total of five ODEs were derived in our calculation.

## Results and discussion

### Batch fermentation

The growth kinetics of *E. mundtii* QU 25 on glucose, cellobiose, and xylose were studied by batch experiments. The batch fermentations were conducted using medium supplemented with varying concentrations of sugars (10, 20, 50, 100 and 150 g L^−1^) and the growth kinetic parameters were determined during the early exponential growth phase with no product inhibition. The optimized values of the kinetic parameters determined by nonlinear regression are listed in Table 1. The maximum growth rates (*μ*_max_) of the studied strain were 1.20 h^−1^, 0.99 h^−1^, and 0.62 h^−1^ for glucose, cellobiose, and xylose, respectively, which were within similar range for other lactic acid-producing strains using different substrates [31–36]. The *μ*_max_ and *K*_*s*_ values for glucose were higher than xylose and cellobiose, implying that glucose is the preferred carbon source of strain QU 25, which was further confirmed by the residual sugars in CSFTR.

**Table 1:** Kinetic parameters from different batch studies and this work (*E. mundtii* QU 25)

Substrates	Microorganism	$\mu_{\text{max} }^{{}}$^a^	$K_{S}^{{}}$^b^	$K_{\text{is}}^{{}}$^c^	$K_{\text{ip}}$^d^	*Y*_la_^e^	*Y*_*X*_^f^	Refs.
		h^−1^	g L^−1^	g L^−1^	g L^−1^	g g^−1^	g g^−1^	
Glucose	*L. amylophilus*	0.32	–	–	–	0.62–0.89	–	[31]
Lactose	*L. plantarum*	0.29	45.0	–	–	0.96	0.25	[32]
Lactose	*L. bulgaricus*	1.14	3.36	119	–	0.90	0.10	[33]
Glucose	*Sporolactobacillus CASD*	0.13	–	–	–	–	–	[34]
Glucose	*L. lactis* NZ133	1.10	1.32	304	1.39	0.93	–	[35]
Molasses	*E. faecealis* RKY1	1.60	0.89	167.46	–	0.9–0.99	0–0.37	[36]
Glucose	*E. mundtii* QU 25	1.20	55.6	108.3	1.8	0.950	0.038	This study
Cellobiose	*E. mundtii* QU 25	0.99	8.9	526.8	1.5	1.015	0.028	
Xylose	*E. mundtii* QU 25	0.62	20.1	254.2	3.3	0.925	0.056	

^a^Maximum specific growth rate; ^b^half saturation concentration; ^c^inhibition coefficient of substrate; ^d^inhibition coefficient of product; ^e^yield of lactic acid production; and ^f^yield of cell production

**Fig. 3:**
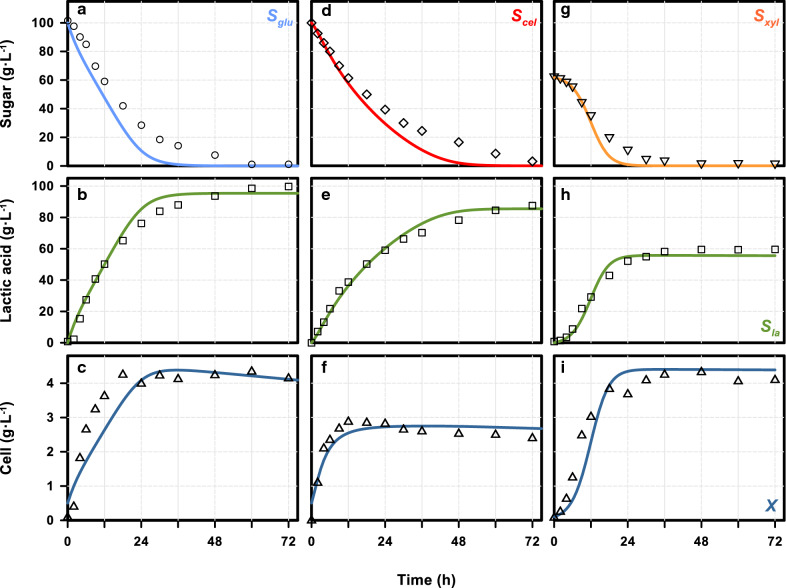
Simulation results (curves) and measurement data (symbols) for lactic acid fermentation by *E. mundtii* QU 25 with different carbon sources, i.e., glucose (left), cellobiose (middle) and xylose (right). The data presented from top to bottom are residual sugars, lactic acid (*LA*), and dry cell weight (*X*). The data points represent the mean values of three independent experiments

Cell growth and lactic acid formation were critically affected by product inhibition (*K*_ip_), while the substrate inhibition (*K*_is_) had relatively small adverse impacts on the studied sugars. A linear relationship was determined between substrate consumption and lactic acid formation with a yield coefficient of 1.0025 for all three sugars (details not shown); this result was similar to the calculated yield of lactic acid based on stoichiometry. The yield of lactic acid was determined from the stoichiometric equations and batch kinetic data with values of 0.950 g g^−1^ for glucose, 1.015 g g^−1^ for xylose, and 0.925 g g^−1^ for cellobiose. The cell yields were in the low range (0.028–0.056 g g^−1^) which could be reasonable if considering the high product yields (0.925‒1.015 g g^−1^). The model parameters were determined by fitting the simulation results with the experimental data. The results of sugar utilization, lactic acid production, and cell growth from three single sugars in the batch system are shown in Fig. 3. After careful adjustment of the parameters, the simulation results (lines) all fit well with the experimental results (symbols). The values of root mean square errors (RMSE), regression coefficient (*R*^2^) bias factor (BF) and accuracy factor (AF) of the model were presented in Additional file 1: Table S1, suggesting a high consistency between the model simulation and experimental results. The validated growth kinetics, such as the maximum growth rate and the half saturation concentrations, were applied in the other simulations for the continuous fermentation processes.

**Fig. 4:**
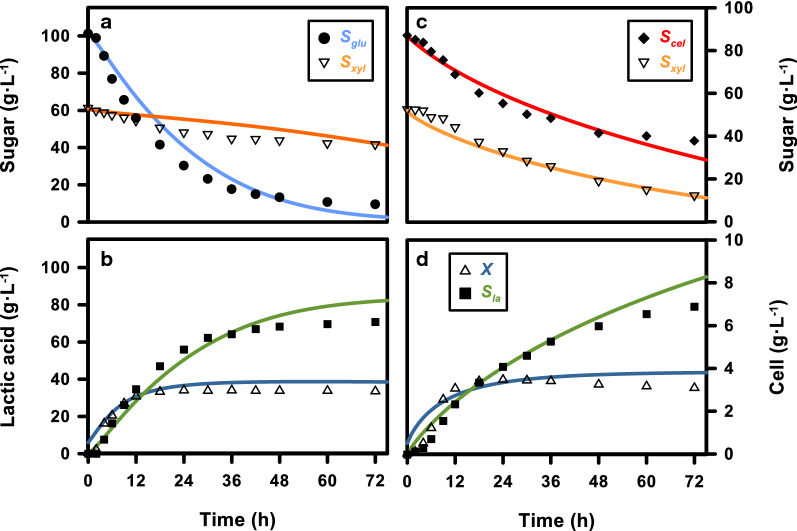
Experiment and simulation results of co-fermentation using various sugar combinations in a batch reactor; carbon catabolite repression was not observed when cellobiose with xylose was mixed. The data points represent the mean values of three independent experiments

In addition, dynamic simulations were also carried out on batch fermentation with mixed sugars (Fig. 4). The impacts of CCR were clearly shown in G100X50 (Fig. 4a), and the majority of xylose was not consumed, as glucose was the preferential carbon source for the synthetic hydrolysate. A large amount of xylose remained in the fermentation broth even when glucose was almost completely converted into lactic acid and cell biomass. On the other hand, CCR was not observed when C100X50 was applied (Fig. 4b) as both sugars were utilized by the fermentation strain simultaneously during fermentation. However, a large amount of cellobiose was not utilized during the testing period when a similar amount of cells was produced in the batch system. The lactic acid concentration was slightly higher in C100X50 than in G100X50.

The simulation results of the dual sugar batch systems showed reasonable fits with the experimental results. The simulation of C100X50 was carried out by the dynamic model without the inclusion of any inhibiting factor from the co-fermenting sugars. To characterize CCR, the simulation of G100X50 was carried out by introducing an inhibiting coefficient from glucose to xylose in Eq. (6). This simulation approach was designed only for this application, as the specific range of glucose concentrations to induce CCR was not clarified. The sensitivity of the inhibiting functions needs to be validated with more comprehensive experiments, which is beyond the scope of this study. As the main focus of this work is to investigate the fermentation of cellobiose and xylose, we tend to study the inhibiting kinetics of CCR in greater detail elsewhere.

### Continuous co-fermentation

This feature of the continuous co-fermentation process for the complete utilization of hexose and pentose was investigated. G100X60 and C100X50 were initially tested at a dilution rate of 0.2 h^−1^ for process control and comparison (Table 2). For G100X60, when continuous fermentation reached a steady state, a cell concentration of 1.99 g L^−1^ was achieved, and a much higher amount of glucose was consumed than xylose (27.5 g L^−1^ over 2.64 g L^−1^, respectively). The ratio of consumed xylose to consumed glucose (X/G) was 0.096, which exhibited an obvious CCR for xylose utilization. *E. mundtii* QU 25 growing on G100X60 produced 24.2 g L^−1^ lactic acid with a yield of 0.803 g g^−1^ and productivity of 4.84 g L^−1^ h^−1^, and a small amount of by-product (0.151 g L^−1^ acetic acid) was detected. When glucose was replaced by cellobiose (C100X60) at the same dilution rate of 0.20 h^−1^, the cell concentration increased to 2.42 g L^−1^ at steady state. A cellobiose consumption of 21.8 g L^−1^ was achieved with a higher xylose consumption of 11.7 g L^−1^, than with G100X50. The ratio of consumed xylose to the consumed cellobiose (X/C) increased to 0.538, suggesting the relief of CCR for xylose consumption. The continuous fermentation process with C100X60 demonstrated a homofermentative lactic acid production of 27.5 g L^−1^ with a yield of 0.820 g g^−1^ and productivity of 5.49 g L^−1^ h^−1^.

**Table 2:** Continuous l-lactic acid production by *E. mundtii* QU 25 using different sugar mixtures in feeding media (G100X60 or C100X60) at dilution rate of 0.2 h^−1^

Mixed sugars^a^	*X*^b^	*S*_glu_^c^	*S*_cel_^d^	*S*_xyl_^e^	X/H^f^ ratios	*S*_la_^g^	*S*_aa_^h^	*Y*_la_^i^	*P*_la_^j^
	g L^−1^	g L^−1^	g L^−1^	g L^−1^		g L^−1^	g L^−1^	g g^−1^	g L^−1^ h
G100X60	1.99 ± 0.05	75.4 ± 1.5	–	57.7 ± 1.3	0.096	24.2 ± 1.8	0.151 ± 0.068	0.803	4.84
C100X60	2.42 ± 0.11	–	79.4 ± 1.7	49.7 ± 0.8	0.538	27.5 ± 0.5	0.383 ± 0.022	0.820	5.49
G100X60	2.06 ± 0.14	72.7 ± 1.0	–	57.6 ± 0.6	0.103	25.4 ± 1.3	0.134 ± 0.017	0.812	5.08

^a^Compositions of fed medium, i.e., G100X60, 100 g L^−1^ glucose and 60 g L^−1^xylose; C100X60, 100 g L^−1^ cellobiose and 60 g L^−1^xylose; ^b^cell concentration; ^c^effluent glucose concentration; ^d^effluent cellobiose concentration; ^e^effluent xylose concentration; ^f^ the ratio of consumed xylose to consumed hexoses (glucose or cellobiose); ^g^lactic acid concentration; ^h^acetic acid concentration; ^i^yield of lactic acid production; and ^j^lactic acid productivity

To confirm the beneficial effects of co-fermentation with C100X60 over that with G100X60, the sugar mixture was changed back to G100X60 after C100X50 was tested. Similar parameters were obtained comparing with the first cycle (Table 2). The sugar combination should be the decisive factor in continuous co-fermentation. Feeding with C100X60 achieved simultaneous sugar utilization in continuous co-fermentation with a high productivity.

### Effects of dilution rates on continuous co-fermentation

The dilution rate is a critical control parameter for maximizing productivity in continuous fermentation process. In this study, continuous co-fermentation of C100X60 was performed at dilution rates increasing from 0.05 to 0.25 h^−1^ (0.05 h^−1^, intervals), and the results are presented in Table 3 (top). The cell formation increased when the dilution rate was between 0.05 h^−1^ to 0.20 h^−1^, and a maximum value of 2.57 g L^−1^ was achieved at 0.20 h^−1^. Further, increasing in the dilution rate to 0.25 h^−1^ decreased cell concentration to 1.74 g L^−1^. Total sugar consumption ranged from 28.5 to 33.7 g L^−1^, and lactic acid production was 20.8–27.9 g L^−1^ at all dilution rates. The X/C ratio was higher than 0.508, among which it increased to 0.543 at a dilution rate of 0.20 h^−1^. The highest productivity of 5.37 g L^−1^ h^−1^ with 26.9 g L^−1^ lactic acid was obtained at a dilution rate of 0.20 h^−1^, which was considered optimal for C100X60 utilization by *E. mundtii* QU 25 in continuous mode. The highest productivity obtained in this study was much higher than 0.635 g L^−1^ h^−1^ in batch experiments [14]. However, high residual cellobiose of 77.2 g L^−1^ and xylose of 50.3 g L^−1^ were observed, and hence, further investigations were carried out to decrease the residual sugars.

**Table 3:** Effect of dilution rates on lactic acid production in CSFTR

	*D*^b^	*X*^c^	*S*_cel_^d^	*S*_xyl_^*e*^	X/C ratio^f^	*S*_la_^g^	*S*_aa_^h^	*Y*_la_^i^	*P*_la_^j^
	h^−1^	g L^−1^	g L^−1^	g L^−1^		g L^−1^	g L^−1^	g g^−1^	g L^−1^ h^−1^
C100X60^a^—CFSTR									
D1	0.05	1.03 ± 0.01	86.6 ± 4.1	44.8 ± 1.4	0.980	25.5 ± 0.3	0.381 ± 0.080	0.887	1.28
D2	0.10	1.43 ± 0.01	82.5 ± 3.8	46.7 ± 2.2	0.671	24.1 ± 0.8	0	0.780	2.41
D3	0.15	2.53 ± 0.03	79.0 ± 1.1	48.1 ± 0.8	0.508	27.9 ± 0.7	0.260 ± 0.039	0.844	4.18
D4	0.20	2.57 ± 0.02	77.2 ± 3.7	50.3 ± 2.5	0.543	26.9 ± 1.2	0.201 ± 0.031	0.823	*5.37*
D5	0.25	1.74 ± 0.05	83.4 ± 1.9	48.2 ± 1.7	0.575	20.8 ± 0.9	0.116 ± 0.023	0.730	5.21
C50X30^a^— CFSTR									
D1	0.05	1.06 ± 0.03	29.3 ± 0.4	20.9 ± 0.4	0.441	32.7 ± 0.5	0.559 ± 0.018	1.09	1.64
D2	0.10	1.43 ± 0.05	28.1 ± 0.5	21.5 ± 0.5	0.412	27.1 ± 1.3	0.380 ± 0.043	0.881	2.71
D3	0.15	1.52 ± 0.08	30.9 ± 1.2	20.7 ± 0.7	0.465	22.1 ± 1.5	0.276 ± 0.058	0.770	3.31
D4	0.20	2.18 ± 0.16	31.3 ± 0.9	22.7 ± 0.2	0.461	22.9 ± 0.9	0.415 ± 0.011	0.871	4.57
D5	0.25	2.42 ± 0.02	26.7 ± 0.4	22.9 ± 0.6	0.304	23.4 ± 0.5	0.651 ± 0.064	0.763	5.85
D6	0.30	2.29 ± 0.08	29.5 ± 0.7	23.7 ± 0.1	0.303	21.7 ± 0.9	0.650 ± 0.031	0.798	*6.52*
D7	0.35	2.16 ± 0.12	30.4 ± 0.5	23.9 ± 0.3	0.312	18.0 ± 0.7	0.570 ± 0.032	0.695	6.29
C50X30^a^— CF/CR									
D0	0.20	33.6 ± 1.9	0.32 ± 0.2	4.71 ± 0.9	0.521	*65.2 ± 3.5*	1.97 ± 0.21	0.854	*13.03*

^a^ Compositions of fed medium, i.e., C100X60, 100 g L^−1^ cellobiose and 60 g L^−1^ xylose; C50X30, 50 g L^−1^ cellobiose and 30 g L^−1^ xylose; ^b^ dilution rates; ^c^cell concentration; ^d^effluent cellobiose concentration; ^e^effluent xylose concentration; ^f^ratios of consumed xylose over consumed cellobiose; ^g^lactic acid concentration; ^h^acetic acid concentration; ^i^yields of lactic acid production; and ^j^lactic acid productivity

C50X30 was investigated in similar fashion as continuous co-fermentation for increasing dilution rates of 0.05–0.35 h^−1^, as presented in Table 3 (bottom). The cell concentration increased with the dilution rate from 1.06 g L^−1^ (at *D *= 0.05 h^−1^) to 2.42 g L^−1^ (at *D *= 0.25 h^−1^), but a further increase in the dilution rates to 0.30 h^−1^ and 0.35 h^−1^ resulted in lower cell concentrations of 2.29 and 2.16 g L^−1^, respectively. The limiting cell retention time (inverse of the dilution rates) of the strain in the CFSTR, or the CRT for zero cell production may be lower than 2 h. The X/C ratios were almost similar at dilution rates of 0.05–0.20 h^−1^ ranged 0.412–0.465, respectively. When the dilution rate was higher than 0.20 h^−1^, the X/C ratio decreased dramatically from 0.461 (at *D *= 0.20 h^−1^) to 0.303 (at *D *= 0.25 h^−1^), implying a critical condition among cell concentration, sugar compositions, and increased CCR for xylose utilization. The lactic acid productivity increased with the increasing dilution rate until *D *= 0.30 h^−1^, and a maximum value of 6.52 g L^−1^ h^−1^ was reached.

**Fig. 5:**
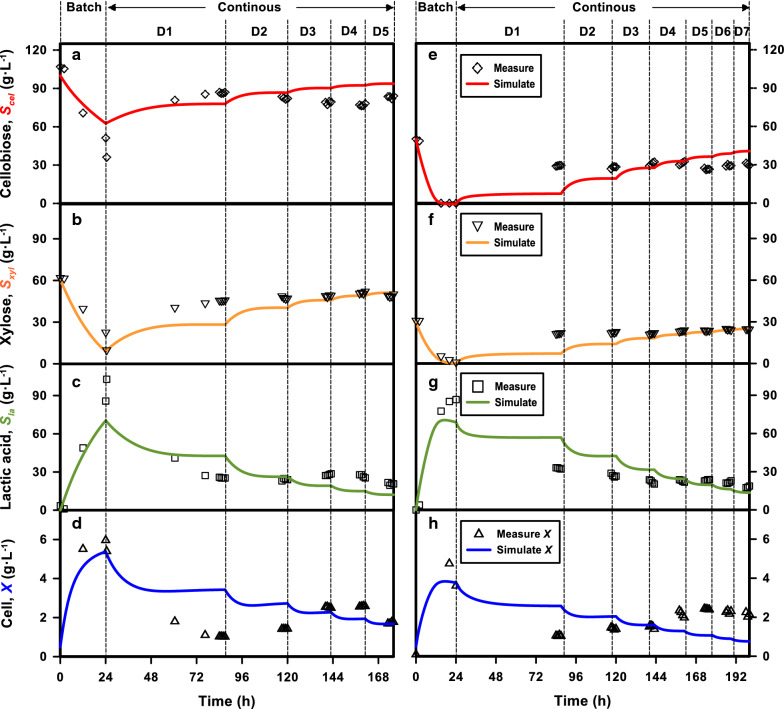
Continuous fermentation to produce lactic acid using *E. mundtii* QU 25 with combined cellobiose and xylose. The designed feeding sugar combinations were cellobiose 100 g L^−1^ and xylose 60 g L^−1^ (C100X60, left); and cellobiose 50 g L^−1^ and xylose 30 g L^−1^ (C50X30, right). Both reactors started with batch mode and then transferred to continuous mode after achieving state state. The color-coded curves show the simulation results and the symbols indicate the experimental results. At least three analyses were carried out before the transition of different operational conditions in the process

As the co-fermentation experiments were carried out continuously, and the influent conditions were adjusted over well-controlled retention time, the experimental results served well as examples of dynamic simulations of the mathematical model. The dynamic records of the experiments and the corresponding simulation results are presented in Fig. 5. Effluent cellobiose, xylose, lactic acid, and cell concentrations are shown in four different rows of the subfigures; and the two columns represent the influent sugars combinations of C100X50 (left) and C50X30 (right). D1–D7 represent the tested dilution rates (0.05–0.35 h^−1^) and the multiple symbols represent the experimental data when steady state was achieved at the tested dilution rates.

In general, the model described reasonably well the dynamic status of the measured parameters, especially on the predictions of immediate changes of substrate and product concentrations between batch and continuous modes. However, some limitations were also discovered for further improvement of the experiments and model structure. In the experiments, cellobiose, xylose, and lactic acid concentrations were at relatively constant levels after the first dilution rate (D1), but obvious increases of cell concentration were observed from D1 to D4 (onward) for both experiments. Before the cell started to be diluted, regional peaks could be found at specific dilution rates, i.e., at the end of D4 for C100X50 and D5 for C50X30. In fact, sugar consumption and lactic acid production also followed this pattern but the covered ranges were less significant. The changes in cell concentrations over dilution rates were not predicted by the simulation.

Based on Monod kinetics, cell concentrations increase with the related consumption of the substrate. This increase is compensated by cell decay and “wash-out” effects due to high dilution rates in CSFTR. As the cell yield coefficients determined in the batch system were quite low, the simulated cell concentration in the process should be a continuously decrease with the increase in dilution rate. This uncertainty between the model and experimental results may be due to the incomplete cell suspension in the fermentation broth or other uncharacterized factors in the model. The fermentor used in this study is a typical cylindrical column container with a mechanical stirrer installed through the reactor from the top. The CSFTR was controlled by pumping the same amount of liquid in and out of the system. The cell samples were collected through a sampling pipe extending to the bottom of the reactor. When performing long-term continuous experiments, the fermenting cells may not be completely suspended in the fermentation broth, or consistently discharged with the liquid effluent. A slightly higher cell concentration may exist in the bottom part of the jar, which results in inconsistency. Regardless, clarification of this issue requires further investigation and was not significant when the fermentation cells were completely retained in the fermentation process, as detailed in the next section.

### Continuous fermentation with cell recycling (CF/CR)

**Fig. 6:**
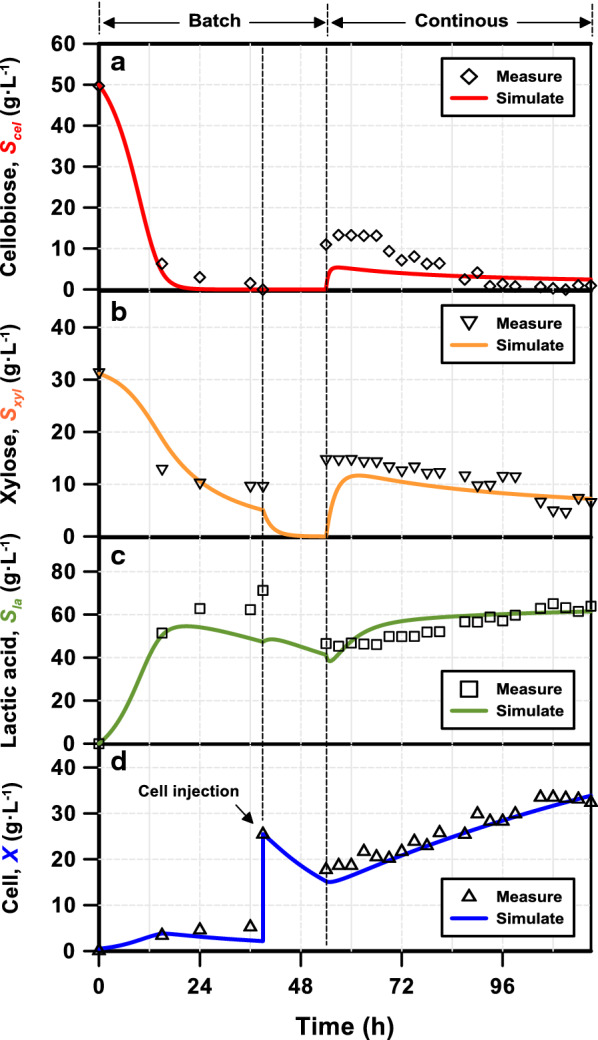
Continuous fermentation with cell recycling to produce lactic acid using *E. mundtii* QU 25 with a combination of cellobiose and xylose. The designed feeding sugar combination was cellobiose 50 g L^−1^ and xylose 30 g L^−1^. The reactor started with batch mode and then transferred to continuous mode after achieving steady state. The cells were concentrated before transfer. The color-coded curves show the simulation results and the points show the averages of three measurements

Controlling cell concentration through cell recycling has been demonstrated to be an efficient technique to obtain high cell density and lactic acid productivity in continuous fermentation with a single carbon source, i.e., glucose [23] and starch [24]. The CF/CR process was performed after receiving concentrated cell concentration from a 4-L reactor, with mMRS medium containing C50X30 at pH 7.0 [14, 21] and a dilution rate of 0.2 h^−1^ (Fig. 6). Approximately 15-fold higher cells (33.6 g L^−1^) and twofold lower residual xylose concentration (4.71 g L^−1^) in the fermentation broth were achieved in comparison to the processes without cell recycling. The X/C ratio was 0.521 in the CF/CR process compared to 0.461 in the conventional mode under the same dilution rate. A high optically pure (≥ 99.8%) l-lactic acid concentration of 65.2 g L^−1^ and productivity of 13.03 g L^−1^ h^−1^ were obtained with slightly lower lactic acid yield over the consumed sugars (0.854 g g^−1^), compared to 22.9 g L^−1^, 4.57 g L^−1^ h^−1^, and 0.871 g g^−1^ without cell recycling, respectively. Only minimal by-products of 0.02–1.97 g L^−1^ acetic acid, 0.26–1.93 g L^−1^ formic acid, and 0–1.65 g L^−1^ ethanol produced from the undesirable pK pathway were measured [7], further confirming our hypothesis in model development. The significant benefits of cell recycle were further clarified when the lactic acid yields were expressed based on the feeding sugars instead of the consumed sugars. In the CF/CR process, almost all the feeding sugars were utilized by the fermentation strain, and the lactic acid yield was 0.801 g g^−1^-feeding sugars; while in the process without cell recycling, the yield of lactic acid was 0.285 g g^−1^-feeding sugars at the same dilution rate of 0.2 h^−1^. The fermentation strategy demonstrated outstanding productivity, end-product concentration, and consumption of mixed sugars for more feasible applications.

The experiment results of the CF/CR process for lactic acid production were compared with the data of the most recent publications in Additional file 1: Table S2. Lactic acid fermentation is a product- and substrate-specific process, and the applied microorganisms and operation conditions play significant roles. The target of the lignocellulosic biomass biorefinery is to increase the product conversion yield from various carbon sources. Continuous fermentation is an attractive concept toward industrialization, and hence has been widely studied recently. The CFSTR operation with free cells suffers from the unbalanced limiting growth rate and metabolic characteristics over short HRTs (high dilution rates), and hence are not feasible in continuous fermentation. Immobilization and cell recycling using hollow fiber microfiltration module have been widely applied to increase the cell density in the bioreactor, and hence further improve the productivity and stability of the process. This concept was confirmed by the experimental results in this study, as the productivity of lactic acid and fermentation titer were both at the high range compared to other studies. Among the references, Tashiro [23] and Ma et al. [25] provided the only two cases with higher productivities than this work. With similar experimental setup, the former study was conducted at an extremely low influent sugar concentration (glucose 20 g/L); and the later one used an outstanding thermophilic strain (*B. coagulans* NBRC 12714) in a well-constructed biorefinery process, i.e., modified pretreatment process and solid background of sequencing fermentation data.

In addition to the productivity, other critical information shown in the table of process performance is the inconsistent relationship between the dilution rate and the production yield. It is widely known that the strain characteristics and experimental setups both play important roles in the fermentation processes, therefore many quantitative measures, i.e., product concentration, yield, productivity, and dilution rate, have been introduced in the studies to support the cross-comparison. However, it should be also noted that the CRT is also a critical parameter but has not been reported throughout the cited literatures. Theoretically, the cell activity is a function of many parameters including the cell aging in the reactor. With the cell metabolism, the productivity and the final product should increase rapidly at the beginning of fermentation, and then reach a plateau before the final decline over time. However, since there is no discharge control of the excessive cells, the CRTs of the reported CF/CR experiments are equal to the running time, which may not directly reflect to this process condition. A standardized index and operational procedure may be needed to support the comparison among the CF/CR studies.

The dynamic change in cellobiose, xylose, lactic acid, and cell concentrations in the CF/CR process (symbols, including the operation in batch mode) and the simulation results (lines) are presented in Fig. 6a through Fig. 6d, respectively. Significant consumptions of cellobiose and xylose were shown at the beginning phase of the batch system (from 0 to 24th h), which was associated with the corresponding increase in lactic acid production and a slightly increase in cell concentration. Xylose was not completely utilized, and approximately 10 g L^−1^ residual sugar in the effluent of the system from 24th to 36th h before cell injection. The concentrated cells were introduced 15 h before the process was changed to continuous mode. The CF/CR functioned properly with consistent reduction in residual sugars and increase in lactic acid/cell concentrations.

The simulation results showed outstanding characterization of the process conditions over the whole experiment. It accurately predicted the consumption of cellulose at batch mode and the overall statuses of the components in continuous mode. While no measurements were conducted during the transition period (from the 39th to the 54th hours), the model simulated the degradation of sugars due to a significant increase in cells, as no additional sugars were introduced in the reactor. During the transition period, the cell concentration declined considerably due to decay, and then increased again in the continuous process when sugars were again introduced in the CF/CR process. Although the CCR on the xylose consumption during the batch mode was not simulated (Fig. 6b, hours 24–36), this model showed high sensitivity in handling the flow condition changes, cell growth, and cell retention problems.

### Importance of cell retention time (CRT)

**Fig. 7:**
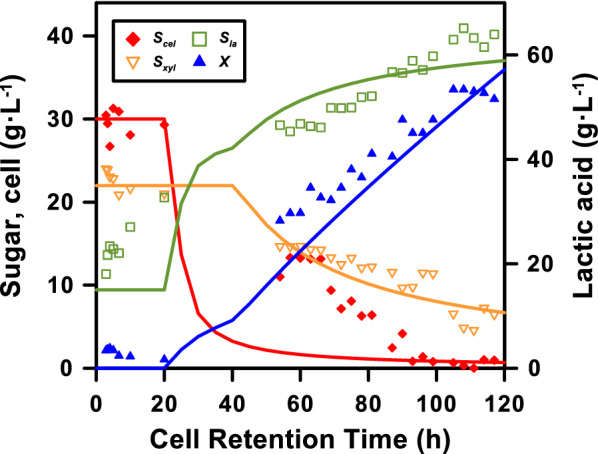
Performances of co-fermentation over different cell retention times (in hours) and two attempted numerical fits: **a** linear regressions; and **b** steady-state expression based on the proposed simulation model

In summary, all the experimental results collected in this study for C50X30, including the relationships of cellobiose, xylose, lactic acid, and cell concentrations, are plotted in Fig. 7. CRTs did show critical impacts on the continuous process. With the increase in CRT, the fermentation strain with a high density was more effective in utilizing the sugars and may be more robust, reflecting the changing properties of the hydrolysate. The benefits of the high cell density fermentation and the CRT control have been demonstrated in many biological systems, i.e., increase in xylose utilization for high biofuel productivity [26], regulation of the consumption rates of various carbon sources [27], and real-time gas-phase monitoring for optimal cells metabolism [28].

Meanwhile, the difference in the commonly applied control parameter dilution rate (1/HRT) used in the conventional fermentation process over the factor for cell retention (1/CRT) should be emphasized. As the cells have been recovered from the liquid stream, the CRT of the CF/CR must be longer than the HRT, and this expression applies to all other biological systems such as bioaugmentation or fed-batch fermentation.

To better visualize the potential applications of the long CRT operation, the dynamic model was simplified to steady-state expressions as the numerical strategy used in Leu et al. [27]. The expressions were derived by eliminating the accumulation terms (i.e., d*X/*d*t* and d*S*/d*t*) in Eqs. (7) and (8), and followed by a series of algebraic operations as presented in Additional file 2: Equations (S1)–(S12). The sugar and cell concentrations as functions of the key control parameters are presented in the following equations:

10 $$S_{\text{sug}} = \frac{{K_{S} \cdot \left( {1 + CRT \cdot K_{D} } \right)}}{{CRT \cdot \left( {\mu_{\text{max} } - K_{D} } \right) - 1}},$$

11 $$X = Y_{\text{sug}}^{X} \cdot \frac{CRT}{HRT} \cdot \frac{{S_{\text{sug}}^{\text{in}} - S_{\text{sug}} }}{{K_{D} \cdot CRT + 1}}.$$

The results of the steady-state expressions were plotted against the experimental results in Fig. 7, which also summarizes the potential benefits and issues of the model. The model clearly demonstrated the possibility of CF/CR operation for process control and optimization. For instance, the important wash-out period at low CRT operation on residual sugars and delayed cell growth was shown in the model, suggesting a potential issue of continuous operation that was not observed in the batch experiments. As the fermentation experiments at short CRT were prepared with the well-inoculated seed strains, the challenges of low CRT operation were not observed in this work. The cell bleeding and complete consumption of xylose at extremely long CRT have not been characterized because the study has targeted and designed experiments for a high productivity. The experiments were discontinued after 117 h of operation when the maximum lactic acid concentration 65.15 g L^−1^ was reached at 108 h and the cell concentration was at the maximum at 105 h. In the simulation model, the cell bleeding and endogenous respiration were all included in the decay term through the first-order reaction kinetics, which may be recorded more clearly if longer and well-controlled CRTs are conducted. The impacts of rapid sugar uptake and delayed growth, or CCR were not characterized due to the current model structure and limited simulation parameters. These uncertain points require further investigations of the system in the future works.

## Conclusion

Continuous co-fermentation was demonstrated to be a feasible strategy to improve the productivity of lactic acid production using a cellobiose/xylose mixture and *Enterococcus mundtii* QU 25. The high productivity 13.03 g L^−1^ h^−1^ and product concentration (65.2 g L^−1^) were contributed by a significant fraction of xylose conversion (34.2%) over cellobiose (65.7%), implying the feasibility of the conversion of lignocellulosic substrates. The kinetic model developed based on the experimental results provided further understanding of the process conditions with various reactor modes, dilution rates, and cell retention times. Further experiments can be designed based on the model structure to clarify the beneficial effects of a long CRT operation for the reduction in CCR and cell decay in the presence of growth-inhibiting substrates.

## Methods

### Microorganism and medium

*E. mundtii* QU 25 was used throughout this study. This strain was found in an ovine fecal sample collected from Fukuoka Zoo, Japan by Dr. Abdel-Rahman and Prof. Sonomoto [15] in our collaborative research team. It is capable to utilize glucose, xylose, and cellobiose for the production of l-(+)-lactate. The strain can produce l-(+)-lactic acid at a high optically purity in the batch (≥ 99.9%) [19] and fed-batch system (≥ 99.7%) [14], and the optimal temperature and pH for culturing were 43 °C and 7.0, respectively [19]. It was confirmed that the strain can carry out the xylose fermentation through the PP/glycolytic pathway instead of the phosphoketolase (PK) pathway. The yield of lactic acid converted from xylose was up to 1.51 mol mol^−1^, which was close to the maximum theoretical yield (1.67 mol mol^−1^) in the PP/glycolytic pathway [19].

The strain sample was stored at ‒80 °C in 2-mL vials containing 15% (v v^−1^) glycerol and refreshed annually. Cell growth, inoculum preparation, and fermentation were conducted using a modified Man, Rogosa, and Sharpe (mMRS) medium containing the following ingredients per liter of distilled water: 10 g peptone (Becton, Dickinson and Company; Sparks, MD, USA), 8 g beef extract (Nacalai Tesque, Kyoto, Japan), 10 g yeast extract (Nacalai Tesque), 2 g K_2_HPO_4_ (Sigma, Tokyo, Japan), 5 g CH_3_COONa·3H_2_O (Nacalai Tesque), 2 g tri-ammonium citrate (Nacalai Tesque), 0.2 g MgSO_4_·7H_2_O (Nacalai Tesque), 0.05 g MnSO_4_·4H_2_O (Nacalai Tesque), and 1 mL Tween 80 (Nacalai Tesque). As indicated later in each experimental description, glucose, xylose (both from Nacalai Tesque), and cellobiose (Carbosynth; Berkshire, UK) were supplemented at several concentrations as carbon sources. In all experiments, the pH of the medium was adjusted to 7.0 by 10 M NH_4_OH [14] or 10 M HCl. The medium and sugar mixtures were sterilized at 115 °C for 20 min separately to prevent heat degradation.

### Hollow fiber microfiltration module

In continuous lactic acid production with cell recycling, a hollow fiber microfiltration module (Microza PMP-102; Asahi Kasei, Tokyo, Japan) was used to recycle cells and feed them back to the fermentor. The filtration area of this module was 0.17 m^2^, every fiber had a diameter of 0.7 mm and a pore diameter of 0.25 μm. Before use, the membrane module was sterilized by 70% ethanol for > 24 h and then washed with sterilized deionized water. The module was washed with sterilized deionized water, and then stored in 1 M NaOH before next experiment.

### Setup of experiments

The fermentation experiments were designed by specific orders to clarify essential metabolic information of *E. mundtii* QU 25 under various conditions, especially the maximum growth rate and decay coefficient. A set of batch fermentations with various sugar combinations was conducted to study the growth kinetics of *E. mundtii* QU 25 and the kinetic parameters were defined. Continuous fermentation was performed after batch cultivation with different dilution rates. A hollow microfiltration fiber was coupled to the fermentation reactor in a closed loop configuration for cell recycling. To confirm the repeatability of the experiments, three independent experiments were carried out for each process condition in batch and continuous modes; and hence, the reported data were the average of triplicates with standard deviation. The CF/CR process was conducted individually, and the reproducibility of the process was verified by three repeated measures of the dynamic change of the process conditions over well-defined frequency (3-h intervals). More details of the experiments are provided in the following sections.

#### Batch and continuous fermentation

All continuous fermentation systems were initiated in a batch mode and then turned into continuous mode after a period of operation. This setup provided us with an opportunity to investigate the changes in microbial activities in the transition phases of the two systems. For inoculum preparation, 1 mL of strain QU 25 glycerol stock was transferred into 9 mL of mMRS medium containing ca. 15 g L^−1^ cellobiose and 15 g L^−1^ xylose and refreshed for 24 h at 43 °C. For pre-culture, 4 mL of the refreshed culture was transferred to a 100-mL flask containing 36 mL of mMRS medium and cultivated for 8 h at 43 °C. Main culture were obtained by inoculating 10% (v v^−1^) pre-cultured broth into a 1-L jar fermentor (Biott; Tokyo, Japan) with a 0.4-L working volume of mMRS medium. pH was controlled at 7.0 with a pH controller (PHC-2201; Able, Tokyo, Japan) by adding 10 M NH_4_OH solution [14]. The process was initiated with batch mode operation at the initial stage for 48 h in ca. 100 g L^−1^ glucose and 60 g L^−1^ xylose (G100X60), 60 h in 100 g L^−1^ cellobiose and 60 g L^−1^ xylose (C100X60), or 36 h in 50 g L^−1^ glucose and 30 g L^−1^ xylose (G50X30). The co-fermentation processes were then switched into continuous mode with designated influent conditions when the majority of the initial sugars were consumed. Samples were taken at steady state at least 4 times at every dilution rate, and analyzed in terms of cell growth and composition of sugars and fermentation products. For all continuous processes, dilution rates were changed after steady state was achieved, or three retention times subsequently tested with the previous dilution rate. The tested dilution rates were 0.05 h^−1^, 0.10 h^−1^, 0.15 h^−1^, 0.20 h^−1^, 0.25 h^−1^, 0.30 h^−1^, and 0.35 h^−1^.

#### Continuous co-fermentation with cell recycling (CF/CR)

Before the continuous co-fermentation, the same refresh and pre-culture processes as previous batch and continuous fermentations were conducted; then strain QU 25 was cultivated initially in batch mode for 36 h in a 5-L jar fermentor containing a 4-L working volume. Once the cell growth reached the late logarithmic growth phase, the broth was gradually transferred to a 1-L fermentor with a pump. Cells were concentrated by recirculation through the hollow fiber module connected to a 1-L jar fermentor. Permeate from the module was collected spontaneously. The fermentation broth was therefore concentrated from 4 L to 0.4 L through this process. The CF/CR process was then initiated with feeding medium containing C50X30 at an agitation rate of 180 rpm with cell recycling. The pH of the broth was maintained at 7.0 by a pH controller with 10 M NH_4_OH. Both the inflow rates of the feeding medium and alkali were balanced to the same rates of outflow of permeates from the module by pumps. Samples were taken at regular intervals.

### Analytical methods

Cell growth was estimated in terms of optical density at a wavelength of 562 nm (OD_562_) with spectrophotometer (UV-1600, Shimadzu). One unit of OD_562_ was equivalent to 0.218 g dry cell weight (DCW, g L^−1^) [19]. The concentrations of cellobiose, xylose, glucose, and fermentation products were analyzed by a high-performance liquid chromatography (HPLC) system (US HPLC-1210, JASCO, Tokyo, Japan) equipped with a refractive index detector (RID) and a SUGAR SH-1011 column (Shodex, Tokyo, Japan) [14]. The collected fermentation samples were centrifuged at 2000×*g* for 10 min at 4 °C, and the supernatant was filtered through a 0.45-μm pore-size membrane filter (Dismic-13HP, Advantec, Tokyo, Japan). HPLC analysis was conducted under the following conditions: injection volume, 20 μL; column temperature, 50 °C; mobile phase, 3 mM HClO_4_; flow rate, 1.0 mL min^−1^ [14]. The optical purity of the produced lactic acid was detected by a BF-5 biosensor (Oji, Hyogo, Japan) based on the manufacturer’s protocol.

The control parameters discussed in this study were as follows: the dilution rate (h^−1^) was calculated as the feed flow rate (mL h^−1^) of the feeding medium divided by the working volume (mL) in the fermentor. Lactic acid productivity (g L^−1^ h^−1^) was calculated as lactic acid produced (g L^−1^) multiplied by the dilution rate (h^−1^). The cell retention time (h) was the average time the fermentation strain stayed in the system, which was equal to the hydraulic retention time (HRT) or the inverse of the dilution rate in CFSTR. When the cell was retained in the reactor, CRT increased with testing time and differed from HRT. In our study, CRT in the cell recycling CFSTR was the same as the overall testing time from the initial stage of the experiments (including the initiation phase operating at batch mode).

### Parameter determination and model validation

The values of the kinetic parameters were determined using the numeric computing program MATLAB R2013a (MathWorks, US). The dynamic functions were solved using the ‘ODE45’ function to simultaneously estimate the kinetic model parameters. The optimization function ‘lsqnonlin’ was adopted for nonlinear least square minimization of the residual between the model estimated values and the corresponding experimental data. The model was validated through searching the most suitable kinetic parameters for the minimum sum of squared differences (SSD) by the Levenberg–Marquardt algorithm. The objective function of minimization to estimate the kinetic parameters is as follows:

12 $$SSD = \text{min} \sum\limits_{i = 1}^{n} {\sqrt {(X_{pi} - X_{\exp i} )^{2} } }$$

where $X_{pi}$ and $X_{\exp i}$ are the model predicted value and experimental data, respectively; *n* is the number of experimental data.

The models were cross-validated against the experimental results from a separated set of single and mixed sugar fermentation. The root mean square error (RMSE), regression coefficient (R^2^), bias factor (BF), and accuracy factor (AF) were calculated to evaluate and validate the flexibility of the models. The RMSE was calculated according to the following equation:

13 $${\text{RMSE}} = \sqrt {\frac{{\sum\limits_{i = 1}^{n} {(X_{pi} - X_{expi} )^{2} } }}{n}} .$$

The BF and AF were applied to evaluate the performance of the proposed kinetic models [29, 30] according to Eqs. (14) and (15).

14 $${\text{BF}} = 10^{{\sum\limits_{i = 1}^{n} {\frac{{\log ({{X_{expi} } \mathord{\left/ {\vphantom {{X_{expi} } {X_{pi} }}} \right. \kern-0pt} {X_{pi} }})}}{n}} }}$$

15 $${\text{AF}} = 10^{{\sum\limits_{i = 1}^{n} {\frac{{|\log ({{X_{pi} } \mathord{\left/ {\vphantom {{X_{pi} } {X_{expi} )}}} \right. \kern-0pt} {X_{expi} )}}|}}{n}} }}$$

The BF and AF equal to 1 indicates a preferred match between the actual observation and model predictions. The AF value is always equal to or greater than 1, and the larger AF value, the less precise the model prediction.

## Supplementary information

**Additional file 1: Table S1.** Validation of mathematical models for sugar consumption, product formation and cell growth. **Table S2.** Comparison of lactic acid production by different continuous fermentation systems.

**Additional file 2:** Step-by-Step Procedure Deriving the Steady-State Expressions of the CF/CR Model.

## Data Availability

All the data have been provided in the manuscript.

## Abbreviations

C50X30: 50 g L^−1^ cellobiose and 30 g L^−1^ xylose
CFSTR: Continuous flow stirring tank rector
CF/CR: Co-fermentation process featured with cell recycle unit
CRT: Cell retention time
*D*: Dilution rate or the inverse of hydraulic retention time (h^−1^)
G100X50: 100 g L^−1^ glucose and 50 g L^−1^ xylose
HRT: Hydraulic retention time (h)
*K*_*D*_: First-order decay coefficient (h^−1^)
*K*_is_: Inhibition coefficient of substrate (g L^−1^)
*K*_ip_: Inhibition coefficient of product (g L^−1^)
*K*_*S*_: Half saturation concentrations (g L^−1^)
*S*_*i*_: Dissolved substrate *i* (g L^−1^)
*X*: Cell concentration (g L^−1^)
X/G: The ratio of consumed xylose to consumed glucose (by mass)
X/C: The ratio of consumed xylose to consumed cellobiose (by mass)
*Y*_*i*_: Molar yield of cell production to the respective consumed substrate_*i*_
$Y_{i}^{j}$: Mass yields of product _*j*_ per unit mass subject _*i*_ consumed (wt %)
_cel_: Cellobiose
_glu_: Glucose
_la_: Lactic acid
_xyl_: Xylose
_max_: Maximum specific growth rate of cell (h^−1^)
