# Correction to: Dynamic simulation of continuous mixed sugar fermentation with increasing cell retention time for lactic acid production using *Enterococcus mundtii* QU 25

**DOI:** 10.1186/s13068-020-01772-2

**Published:** 2020-08-09

**Authors:** Ying Wang, Ka-Lai Chan, Mohamed Ali Abdel-Rahman, Kenji Sonomoto, Shao-Yuan Leu

**Affiliations:** 1 Department of Biological Science, College of Life Sciences, Sichuan Normal University, Chengdu, 610101 Sichuan China; 2 Department of Civil and Environmental Engineering, Hong Kong Polytechnic University, Kowloon, Hong Kong; 3 Laboratory of Microbial Technology, Division of Systems Bioengineering, Department of Bioscience and Biotechnology, Faculty of Agriculture, Graduate School, Kyushu University, Motooka, Nishi‐ku, Fukuoka, Japan; 4 Botany and Microbiology Department, Faculty of Science (Boys), Al-Azhar University, PN:11884, Nasr City, Cairo, Egypt

## Correction to: Biotechnol Biofuels (2020) 13:112 10.1186/s13068-020-01752-6

Following publication of the original article [1], the authors identified an error in Fig. 1. The correct figure is given below.

**Fig. 1:**
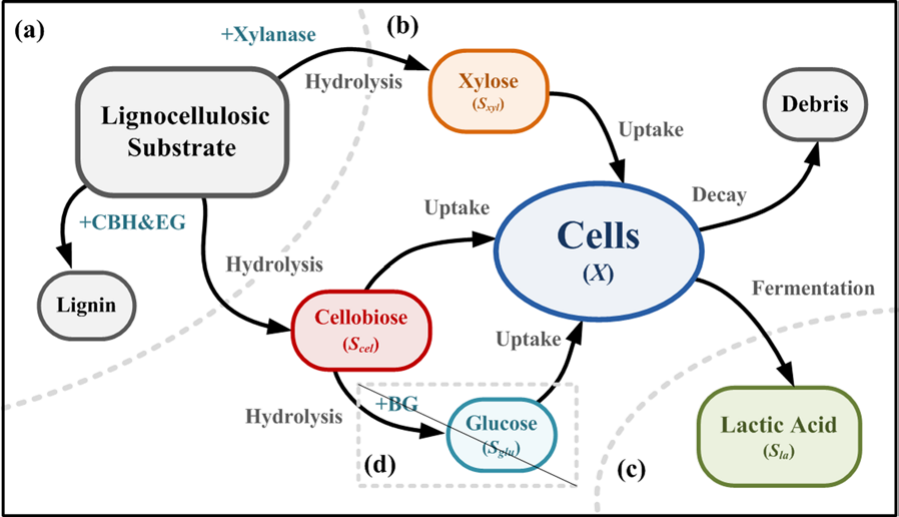
Conceptual diagram and model structure of the continuous lactic acid co-fermentation process with membrane separation and selected enzyme combination for preventing carbon catabolite repression; **a** hydrolysis system; **b** fermentation; **c** membrane separation; and **d** pathway to be cut-off to prevent carbon catabolite repression (CCR)

The original article has been corrected.
