# Supplementary material for: Dynamic simulation of continuous mixed sugar fermentation with increasing cell retention time for lactic acid production using Enterococcus mundtii QU 25
Source: Biotechnol Biofuels. 2020 Jun 26;13:112. doi: 10.1186/s13068-020-01752-6 (PMC7318410; doi:10.1186/s13068-020-01752-6)
Supplement: Supplementary file 1 — Additional file 1: Table S1. Validation of mathematical models for sugar consumption, product formation and cell growth. Table S2. Comparison of lactic acid production by different continuous fermentation systems. [file 13068_2020_1752_MOESM1_ESM.docx]

**Supplementary information**

**Table S1.** Validation of mathematical models for sugar consumption, product formation and cell growth.

| Substrates | Sugar consumption | | | |  | Production formation | | | |  | Cell growth | | | |
| --- | --- | --- | --- | --- | --- | --- | --- | --- | --- | --- | --- | --- | --- | --- |
|  | RMSE^a^ | R^2b^ | BF^c^ | AF^d^ |  | RMSE | R^2^ | BF | AF |  | RMSE | R^2^ | BF | AF |
| Glucose | 12.6 | 0.964 | 1.20 | 1.20 |  | 5.29 | 0.981 | 0.88 | 1.26 |  | 0.71 | 0.865 | 1.05 | 1.27 |
| Cellobiose | 7.99 | 0.967 | 1.08 | 1.08 |  | 3.17 | 0.992 | 1.04 | 1.09 |  | 0.21 | 0.930 | 1.03 | 1.08 |
| Xylose | 4.40 | 0.990 | 1.23 | 1.23 |  | 3.83 | 0.995 | 1.01 | 1.11 |  | 0.23 | 0.978 | 0.93 | 1.20 |

^a^ root mean square error; ^b^ regression coefficient; ^c^ bias factor; ^d^ accuracy factor.

**Table S2.** Comparison of lactic acid production by different continuous fermentation systems.

| Fermentation Mode | Substrates | Microorganism | *S*_la_^a^  (g·L^-1^) | *Y*_la_^b^  (g·g^-1^) | *P*_la_^c^  (g·L^-1^·h^-1^) | Isomer (optical purity, %) | *D*^d^  *(h^-1^)* | Ref. |
| --- | --- | --- | --- | --- | --- | --- | --- | --- |
| Free cells | Corn stover hydrolysate | *B. coagulans* strain AD | 42 | 0.95 | 3.69 | ND^e^ | 0.167 | [[37](#_ENREF_37)] |
| Free cells | Lactose: 60 g·L^-1^ | *Lb. bulgaricus* ATCC 8001 | 30 | 0.72 | 7.2 | ND^e^ | 0.24 | [[38](#_ENREF_38)] |
| Free cells | Xylose: 50 g·L^-1^ | *E. mundtii* QU 25 | 21.7 | 0.855 | 3.14 | l (-) | 0.15 | [[39](#_ENREF_39)] |
| Free cells | C50X30^f^ | *E. mundtii* QU 25 | 21.7 | 0.801 | 6.52 | l (99.8) | 0.3 | This work |
| Immobilization | Glucose: 45 g·L^-1^ | *Lc. lactis* IO-1 | 8.91 | 0.89 | 4.46 | ND^e^ | 0.5 | [[40](#_ENREF_40)] |
| Immobilization | Lactose: 100 g·L^-1^ | *Lb. helveticus* ATCC 15009 | 75.6 | 0.96 | 3.90 | ND^e^ | 0.056 | [[41](#_ENREF_41)] |
| Cell recycling | Glucose: 20 g·L^-1^ | *Lb. delbrueckii subsp. lactis* QU 41 | 20.7 | 1.03 | 18.0 | d (99.9) | 0.87 | [[23](#_ENREF_23)] |
| Cell recycling | Corn steep: 30 g·L^-1^ | *Lactobacillussp.* RKY2 | 42.0 | 0.95 | 6.7 | ND^e^ | 0.16 | [[42](#_ENREF_42)] |
| Cell recycling | Tapioca hydrolysate | *B. coagulans* A107 | 50.3 | 0.80 | 10.1 | l (+) | 0.2 | [[43](#_ENREF_43)] |
| Cell recycling | Acid whey: 90 g·L^-1^ | *L. coryniformissubsp.torquens* | 45.9 | 0.92 | 9.2 | l (-) | 0.2 | [[43](#_ENREF_43)] |
| Cell recycling | Molasses | *B. coagulans* A40 | 59.6 | 0.85 | 5.9 | l (+) | 0.1 | [[43](#_ENREF_43)] |
| Cell recycling | Corn stover hydrolysate | *B. coagulans* NBRC 12714 | 92 | 0.91 | 13.8 | l (99.5) | 0.15 | [[25](#_ENREF_25)] |
| Cell recycling | Glucose: 50 g·L^-1^ | *B. coagulans* PS5 | 42 | 0.84 | 8.6 | l (-) | 0.2 | [[44](#_ENREF_44)] |
| Cell recycling | Glucose: 100 g·L^-1^ | *E. faecalis* RKY1 KCTC 8890P | ≥90 | 0.95 | 3.72 | l (+) | 0.04 | [[45](#_ENREF_45)] |
| Cell recycling | Xylose: 50 g·L^-1^ | *E. mundtii* QU 25 | 41.0 | 1.01 | 6.15 | l (-) | 0.155 | [[39](#_ENREF_39)] |
| Cell recycling | C50X30^f^ | *E. mundtii* QU 25 | 65.2 | 0.854 | 13.03 | l (99.8) | 0.2 | This work |

^a^Lactic acid concentration. ^b^Yield of lactic acid production. ^c^Lactic acid productivity. ^d^Dilution rate. ^e^Not determined. *Lb., Lactobacillus*; *Lc., Lactococcus*；*E., Enterococcus; B., Bacillus;* and ^f^Cellobiose 50 g·L^-1^ and xylose 30 g·L^-1^.
