# Supplementary material for: Dynamic simulation of continuous mixed sugar fermentation with increasing cell retention time for lactic acid production using Enterococcus mundtii QU 25
Source: Biotechnol Biofuels. 2020 Jun 26;13:112. doi: 10.1186/s13068-020-01752-6 (PMC7318410; doi:10.1186/s13068-020-01752-6)
Supplement: Supplementary file 2 — Additional file 2: Step-by-Step Procedure Deriving the Steady-State Expressions of the CF/CR Model. [file 13068_2020_1752_MOESM2_ESM.docx]

**Step-by-Step Procedure Deriving the Steady-State Expressions of the CF/CR Model**

This document provides the step-by-step procedure reforming the dynamic bioreactor model into the steady-state expressions in the continuous flow co-fermentation system with cell recycling control (the CF/CR process, **Fig.S1**). The fermentation cells (*X*) and sugar (*S_sug_*) concentrations were presented in functions of two key control parameters, *i.e*., the hydraulic retention time (*HRT*) and the cell retention time (*CRT*). The calculation was developed in a similar fashion as presented in Leu et al. [1], based on the Monod kinetics and Lawrence and McCarty expressions [2] as follow:

For cells:

$\frac{dX}{dt}=-\frac{Q_{W}\cdot X_{W}}{V}+(\mu-K_{D})\cdot X$ (S1)

For sugar:

$\frac{dS_{sug}}{dt}=\frac{1}{HRT}\cdot\left(S_{sug}^{in}-S_{sug}\right)-\frac{\mu}{Y_{sug}^{X}}\cdot X$ (S2)

where *Q_W_* is the wasted flow rate of the discharged cell (L∙h^-1^); *X_E_* and *X_W_* are effluent and wasted cell concentration (g∙L^-1^); *μ* is the growth rate of the cell (h^-1^). The rest of the nomenclature please refer to the main text.

At steady state, *dX/dt* and *dS_sug_/dt* = 0

From Equation (c1)

$\mu-K_{D}=\frac{Q_{W}\cdot X_{W}}{X\cdot V}$ (S3)

From Equation (c2)

$\mu\cdot\frac{X}{Y_{sug}^{X}}=\frac{1}{HRT}\cdot\left(S_{sug}^{in}-S_{sug}\right)$ (S4)

By definition

$CRT=\frac{X\cdot V}{Q_{W}\cdot X_{W}}$ (S5)

Therefore,

$\mu-K_{D}=\frac{1}{CRT}$ (S6)

Substitute *μ* in (c4) with (c6)

$\frac{1}{Y_{sug}^{X}}\cdot\left(K_{D}+\frac{1}{CRT}\right)=\frac{1}{HRT}\cdot\frac{\left(S_{sug}^{in}-S_{sug}\right)}{X}$ (S7)

Therefore,

$X=Y_{sug}^{X}\cdot\frac{CRT}{HRT}\cdot\left(\frac{S_{sug}^{in}-S_{sug}}{K_{D}\cdot CRT+1}\right)$ (S8)

With Monod kinetics

$\mu=\frac{\mu_{\max}\cdot S_{sug}}{K_{S}+S_{sug}}$ (S9)

Apply Equation (a9) to Equation (a6)

$\frac{\mu_{\max}\cdot S_{sug}}{K_{S}+S_{sug}}-K_{D}=\frac{1}{CRT}$ (S10)

After rearrangement

$S_{sug}=\frac{K_{S}\cdot(1+CRT\cdot K_{D})}{CRT\cdot(\mu_{\max}-K_{D})-1}$ (S11)

Equation (S8) and Equation (S11) were used as Equation (10) and Equation (11) in the manuscript, respectively.

In a short CRT process or CSFTR process operating at high dilution rate, the limiting cell retention time occurred when *$CRT\cdot(\mu_{\max}-K_{D})-1\leq0$*, so the critical CRT will be:

$CRT=\frac{1}{\mu_{\max}-K_{D}}$ (S12)

The critical condition resulted in no production of cells or lactic acid at short CRT as hour-0 to hour-20 in the simulation presented in **Fig.7**.

**References**

1. Leu, S.Y., et al., *Modeling the performance of hazardous wastes removal in bioaugmented activated sludge processes.* Water environment research, 2009. **81**(11): p. 2309-2319.

2. Lawrence, A.W. and P.L. McCarty, *Unified basis for biological treatment design and operation.* Journal of the Sanitary Engineering Division, 1970. **96**(3): p. 757-778.
